# Understanding social engagement in autism: being different in perceiving and sharing affordances

**DOI:** 10.3389/fpsyg.2014.00850

**Published:** 2014-08-04

**Authors:** Annika Hellendoorn

**Affiliations:** Department of Special Education, Centre for Cognitive and Motor Disabilities, Utrecht UniversityUtrecht, Netherlands

**Keywords:** social cognition, theory of mind, embodied cognition, affordances, autism spectrum disorder

## Abstract

In the current paper I will argue that the notion of affordances offers an alternative to theory of mind (ToM) approaches in studying social engagement in general and in explaining social engagement in autism spectrum disorder (ASD) specifically. Affordances are the possibilities for action offered by the environment. In contrast to ToM approaches, the concept of affordances implies the complementarity of person and environment and rejects the dualism of mind and behavior. In line with the Gibsonian idea that a child must eventually perceive the affordances of the environment for others as well for herself in order to become socialized, I will hypothesize that individuals with ASD often do not perceive the same affordances in the environment as other people do and have difficulties perceiving others’ affordances. This can lead to a disruption of interpersonal behaviors. I will further argue that the methods for studying social engagement should be adapted if we want to take interaction into account.

How people are able to interact successfully with each other is a question raised and answered by researchers from different disciplines. While this question can be answered in numerous ways, the answer that emerges from a significant part of the literature is by employing a “Theory of Mind” (ToM). Although there are different definitions of this concept, the term “ToM” generally refers to the ability to attribute mental states to the self and other people in order to explain and predict behavior (Premack and Woodruff, 1978; Baron-Cohen et al., 2000). ToM approaches assume that people have a ToM that enables them to *infer*, either explicitly or implicitly, the mental state of a person from that person’s behavior (Van Overwalle and Vandekerckhove, 2013). This implies that ToM theory separates the (supposedly meaningless) observable behavior from the (meaningful) private mind in a Cartesian way and ToM approaches have been criticized for that way of thinking (Gallagher, 2004; Reddy, 2008; Leudar and Costall, 2009/2011). From this perspective you need a ToM in order to interact successfully with other people. In addition to the criticism of Cartesian dualism, ToM approaches have also been criticized for isolating social understanding from the actual engagement (De Jaegher and Di Paolo, 2007; Fuchs and De Jaegher, 2009). According to ToM approaches, meaning is constructed in the minds of social participants. The idea that meaning is created in the ongoing active interaction between persons is not taken into account (Fuchs and De Jaegher, 2009).

In contrast to ToM approaches, more embodied approaches assume that mind and behavior are not separate. People directly perceive other persons’ intentions in their actions without the need for an indirect, implicit or explicit, process of inference and theory (Gallagher, 2001, 2004; Good, 2007). This is consistent with the concept of “affordances.” Affordances are the action possibilities that the environment offers to an animal or person (Gibson, 1986). It is assumed that affordances are perceived directly, i.e., without the intervention of certain cognition operations, such as ToM (Gibson, 1986; Barrett, 2011). Directly does not mean that every affordance in the environment is automatically perceived and acted upon. The perception of affordances is dependent upon the particular information that is picked up by the perceiver and the information pick-up is dependent upon the characteristics and capabilities of the perceiver (e.g., the central nervous system, perceptual system, motor skills) and the interaction that the perceiver has with the environment (Gibson and Pick, 2000). Thus, an affordance is inherently specific to a particular perceiver. What an object or the action of another person affords one person may be different from what the same object or action affords someone else. What is relevant in the environment cannot be separated from the perceiver, it is not a pre-given. In addition to individual differences, different groups may also show differences in the perception of affordances. Differences have been found between novices and experts (Charness et al., 2001), between children with different motor skills (Adolph et al., 1993), between children and adults (Thelen, 2008), and between typically developing persons and persons with a physical or mental impairment (Loveland, 1991).

Gibson (1986) explicitly states that the perception of social affordances, which may be defined as the affordances provided by other people’s behavior, is just as direct and based on the pick-up of information as the perception of affordances in the physical environment: “It is just as much based on stimulus information as is the simpler perception of support that is offered by the ground under one’s feet. For animals and other persons can only give off information about themselves insofar as they are tangible, audible, odorous, tastable and visible” (Gibson, 1986, p. 135). However, social affordances are also different from affordances of the physical environment: “They are so different from ordinary objects that infants learn almost immediately to distinguish them from plants and non-living things. When touched they touch back, when struck they strike back; in short they interact with the observer and with one another. Behavior affords behavior…” (Gibson, 1986, p. 135). This means that the actions of persons in social interaction are not only dependent upon the attunement (the particular information that is picked up) of both persons individually, but their actions are also dependent on the action of the other person. “What the infant affords the mother, is reciprocal to what the mother affords the infant” (Gibson, 1986, p. 135). Thus, social affordances are actively created and maintained by the joint action of two or more persons (Good, 2007). This is consistent with the idea that interactors’ perception-action loops are coupled and interlaced with each other and that in social interaction agents participate in each other’s sense-making (De Jaegher and Di Paolo, 2007; Fuchs and De Jaegher, 2009).

## UNDERSTANDING SOCIAL ENGAGEMENT IN AUTISM

It has been claimed that ToM theory can explain the social-communicative impairments of autism spectrum disorders (ASD; Baron-Cohen et al., 1985): “We have reason to believe that autistic children lack such a “theory.” If this were so, then they would be unable to impute beliefs to others and to predict their behavior” (Baron-Cohen et al., 1985, p. 37). I have introduced the concept of social affordances as an alternative to ToM. Since affordance perception is based on the pick-up of information, the explanation for the social-communicative impairments in ASD from an ecological perspective should be sought in differences in information pick-up between people with and without ASD and the cascading effects this will have for the interaction. Several theories and studies have indicated that both children and adults with ASD pick-up different information compared to people without ASD (Mottron et al., 2006; Gepner and Féron, 2009; De Jaegher, 2013; Donnellan et al., 2013). An example might be emotion perception. Emotions can be viewed as social affordances in the sense that they call forth various interpersonal behaviors. For example, anger is likely to provoke avoidance, whereas joy is likely to encourage approach (McArthur and Baron, 1983). Studies show that the information that specifies facial expressions is a specific spatial integration of different facial features changing in a characteristic way. Perceivers respond to changes in the whole facial configuration. That information is critical and sufficient for face recognition and emotion perception (Tanaka et al., 1998; Behrmann et al., 2006a; Pellicano et al., 2006), and is largely supported by low spatial frequency information (Goffaux and Rossion, 2006). Studies indicate that individuals with ASD are less sensitive to configurations than people without ASD and show enhanced sensitivity in response to high spatial frequency (fine perceptual detail, sharp edges) versus low spatial frequency (general shape and large contour) stimulus information, compared to typically developing and developmentally delayed children and both for neutral as well as socially relevant stimuli (Deruelle et al., 2004; Vlamings et al., 2010). This is in accordance with personal accounts: “*I did not see the whole. I saw hair, I saw eyes, nose, mouth, chin, … not face*.” (Alex in Williams, 1999, p. 180). These studies suggest that the facial expression may not afford the “typical” social behavior for people with ASD, because the facial expression, specified by configural information, may be difficult to perceive for persons with ASD. Studies on biological motion support the idea that affordances are specified by a particular type of information that is detected by typically developing individuals, but not by individuals with ASD. Johansson (1973) has designed experiments in which a few spots show the motions of the main joints of a person. When a moving presentation of this minimal information is shown to typically developing persons they can tell whether the point-light display is walking, dancing, fighting, etc. Studies show that children with autism have difficulties recognizing biological motion and emotion from point-light displays, while typically developing children and children with spatial deficits and a degree of mental retardation are able to do that (Jordan et al., 2002; Blake et al., 2003; Annaz et al., 2010; Nackaerts et al., 2012). Children with ASD also show a different pattern of eye movements while seeing point-light displays (Nackaerts et al., 2012). Other studies that have tested information pick-up through eye-tracking methods confirm that there are clear differences in information pick-up between people with and without ASD (Klin et al., 2002). This means that what a situation affords for a person with ASD is often different from what the same situation affords for a personwithout ASD.

In addition, as stated before, behavior affords behavior. Therefore the different information pick-up of a person with ASD will not only affect the actions of that person, but also the actions of the other person(s) in the interaction. Typically, although there may be many individual differences in the affordances that people perceive, there is some common ground in the sense that persons that are somewhat similar in capabilities, experience and culture perceive the same affordances in social interaction, i.e., they will act alike in a similar social context. However, it is well-known that a person with ASD will often act differently than a person without ASD in the same context, both in relation to the physical and to the social environment. Gibson (1986) notes that in order to become socialized a child has to perceive the affordances for herself as well as for others. In an interaction between a person with and without ASD, the dyadic partners may not be able to perceive the affordances of the other person and this may disrupt the rhythm of interaction. Trevarthen and Daniel (2005) have for instance shown that parents of children with ASD have difficulties in engaging with their child and that these interactions are characterized by less rhythmic interaction. In triadic interactions involving an object, the fact that two partners perceive different affordances of the object may also lead to less smooth interactions. Preference for producing or observing spinning or rotating movements (spinning objects, watching washing machine rotating, spinning wheels of toy cars) is for instance common in children with ASD (Bracha et al., 1995). If one child is for instance continuously spinning the wheels of a toy car while the other child is “driving” the car, this will probably decrease the amount of social interaction between the children. Consistent with the idea that a different affordance perception in ASD may underlie their social-communicative impairments, several studies have indicated that disturbances in basic perception-action process may underlie and are related to social-communicative impairments (Mottron et al., 2006; Gepner and Féron, 2009; De Jaegher, 2013; Donnellan et al., 2013; Kapp, 2013; Hellendoorn et al., 2014).

Although it should be taken into account that ASD is a pervasive developmental disorder which affects many developmental domains (Yirmiya and Charman, 2010), it is important that it is explained why there are *more* differences in the perception of social affordances between people with and without ASD than in the perception of affordances in the physical environment. Gibson (1986) already notes that: “The richest and most elaborate affordances of the environment are provided by other people. They move from place to place, changing the postures of their bodies… . The perceiving of these mutual affordances is enormously complex” (p. 135). Thus, there may be two explanations as to why social-communicative impairments are so pronounced in individuals with ASD. First of all, the perception of social affordances is different from the perception of affordances of objects because of *the nature of* social information. The social information consists of many features, is dynamic and multimodal (McArthur and Baron, 1983). Several studies show that children with ASD have specific difficulties, both delays and impairments, with perceiving dynamic and configural information, also in non-social situations (Deruelle et al., 2004; Behrmann et al., 2006b; Gepner and Féron, 2009; Annaz et al., 2010; Vlamings et al., 2010; Weisberg et al., 2014). This implies that the differences in perceiving social affordances between people with and without ASD cannot be attributed to the fact that the information is *social per se*, but to the fact that a lot of social affordances are specified by information that is difficult to pick-up for people with ASD. Another reason that may explain why people with ASD have the most difficulties in the social-communicative domain, may be related to the aforementioned idea that the different affordance perception of a person with ASD has cascading and possibly disrupting effects for the whole interaction since social affordances are actively created and maintained by the joint action of the actors in the interaction (Good, 2007). Thus, the affordances in the interaction between a person with ASD and a person without ASD will be different than the affordances in the interaction between two persons without ASD. It may even be that the social interaction affords a person with ASD to disengage, because the different perception of affordances makes the interaction very unpredictable, uncontrollable and stressful for them. Without interaction with other persons, the person with ASD will never learn to perceive the affordances and moreover, disengagement prevents the creation of affordances in interaction with other persons.

For some people with ASD social interaction affords a kind of disengagement in the sense that they explicitly theorize about social interaction, instead of engaging (Williams, 2004). Below are a few examples of how high-functioning people with ASD describe what they are doing in the social environment: “I was a scientist trying to figure out the ways of the natives. I wanted to participate, but I didn’t know how” (Grandin, 1996, p. 132; cited in Williams, 2004). “By studying an individual’s posture, actions, voice tone, and expression, I can now usually work out what they are feeling.” (Lawson, 2001, pp. 8–9; cited in Williams, 2004). The fact that high-functioning individuals with ASD can and do reason about social behavior does not imply that persons with ASD use ToM-style operations. In contrast, the fact that people with ASD act in this way in social interaction, while those that develop typically do not to theorize about social behavior in most social interactions, may actually show that they do not perceive the same affordances in a social environment. While the social environment affords engagement for the typically developing persons it affords detached theorizing for these high-functioning persons with ASD. As Reddy (2008) notes any “theory theory” is a very different understanding than skilled interaction with the environment: more like the understanding of a bystander than that of participant. This is also supported by studies that show that the performance of persons in ToM-like operations is not related to the skills people have in real-life engagement with other persons (Ozonoff and Miller, 1995).

## TOWARD DIFFERENT RESEARCH METHODS

Investigating social competence from an affordance approach requires different research methods than the methods that have been used to investigate social skills within a ToM paradigm. Research within the affordance approach should provide us with a description of the information people are responding to in social interaction, i.e., a description of which information people use to inform their actions. People with typical and atypical development, but also for instance children and adults, could then be compared to examine whether there are differences in the information they pick-up and use in social interaction. While the study of Klin et al. (2002) already provides an interesting example of such a design comparing people with and without autism with regard to their focus of attention while viewing a social scene, the participants in that study were still rather passive and detached from the interaction because the social scenes they watched were displayed on a video screen, and it was not an immersed situation. In line with the idea that cognition emerges in the interaction in a continuous perception-action cycle wherein behavior affords behavior, a study design with mobile eye tracking and coding of behavior of all participants in a real-time social interaction could provide the data that fits within the affordance approach to social perception. Since the actions of one person shape the actions of the other person (i.e., behavior affords behavior) more attention should also be given to research methods that measure variables of the interaction (*inter*-personal variables) instead of only focusing on *intra*- personal variables. De Jaegher (2006) states for instance that timing is a foundational aspect of successful social interaction which is disturbed in ASD.

In conclusion, “overcoming the myth of the mental,” as Dreyfus (2006) states it, is difficult as is indicated by the popularity of ToM approaches and other approaches that fit within a cognitivist tradition. An embodied ecological perspective may offer a fruitful alternative to these approaches for studying both social and non-social cognition. The concept of affordances does justice to the idea that mind and behavior cannot be separated. People with ASD are not attuned to the same information as people without ASD. This leads to the specification of different affordances and may have cascading effects for the interaction with other persons. In conclusion, not only do people with autism experience or understand the world differently from other people, the environment (including other persons) really affords different behavior, simply because they are in it (Loveland, 2001).

## Conflict of Interest Statement

The author declares that the research was conducted in the absence of any commercial or financial relationships that could be construed as a potential conflict of interest.
